# H Syndrome: Report of The First Case in African Ethnicity

**DOI:** 10.7759/cureus.23281

**Published:** 2022-03-17

**Authors:** Osama Khder Ahmed Elmansour, Ahmed Osama Ahmed Babikir

**Affiliations:** 1 Department of Internal Medicine, Shendi University, Faculty of Medicine, Shendi, SDN; 2 Department of Pathology, Shendi University, Faculty of Medicine, Shendi, SDN

**Keywords:** inflammatory arthritis, genodermatosis, human equilibrative transporter 3, slc29a3 gene, h syndrome

## Abstract

H syndrome is an autosomal recessive multisystemic disease with a very low prevalence rate, characterized by indurated cutaneous hyperpigmentation, hypertrichosis, and various systemic manifestations. The syndrome is caused by mutations in SLC29A3 gene on chromosome 10q23, encoding for human equilibrative transporter 3 (hENT3). So far, only 100-120 patients with H syndrome have been described in the literature, with predominance among Indian, North-American, and Arab ethnicities.

This case report describes the first one of H-syndrome rarities in African ethnicity, a 30-year-old Sudanese male misdiagnosed with rheumatoid arthritis. The patient exhibited more than 90% of the clinical characteristics of H syndrome including obesity, short stature, characteristic hyperpigmented, sclerotic cutaneous plaques with induration and hypertrichosis, inflammatory arthropathy, hallux valgus, flexion deformity of toes, exophthalmos, cardiac anomaly, hypogonadism, and splenomegaly and characteristic histologic findings of dermal fibrosis, histiocytosis, lymphoid aggregation, and vascular proliferation.

H syndrome is an extremely rare autoinflammatory condition that has a complex constellation of pleiotropic manifestations with multisystemic involvement. And while further identification and better pathophysiological understanding of H syndrome are needed, physicians worldwide should be vigilant about the overlapping features of H syndrome with many other rheumatological, cutaneous, and genetic diseases.

## Introduction

H syndrome is a rare autosomal recessive, multisystemic, autoimmune inflammatory disorder caused by mutations in the SLC29A3 gene on chromosome 10q22, encoding the human equilibrative nucleoside transporter 3 (hENT3) [1]. The condition comprises a constellation of various symptoms and clinical signs, with characteristic cutaneous hyperpigmentation, hypertrichosis, and induration being the pathognomonic feature of the disease [2]. Other manifestations of H syndrome include cardiac anomalies, histiocytosis, hepatosplenomegaly, sensorineural hearing loss, exophthalmos, insulin-dependent diabetes mellitus, genital abnormalities (hypogonadism), and skeletal deformities (fixed flexion contractures of proximal interphalangeal joints).

Since first reported in 2008 [1], H syndrome is considered to be a very rare condition to encounter with only 100 cases reported in world literature [3]. The defective gene in H syndrome (SLC29A3) encodes the hENT3. And while multiple mutations have been denoted, it is well comprehended now that hENT3-related spectrum disorders share a common mutation with vast and overlapping clinical and multisystemic manifestations, as this transporter is involved with passive sodium-independent transportation of nucleosides and considered critical for nucleotides synthesis via salvage pathways [4].

## Case presentation

We report the case of a 30-year-old Sudanese male born of a second-degree consanguineous marriage presenting to our causality with generalized fatigability, malaise, fever, and multiple painful, tender joints. The patient’s condition started as early as 14 years prior to presentation, with him developing painless, indurated, well-circumscribed, non-pruritic areas of hyper-pigmentation and marked hypertrichosis limited in the upper medial aspect of the thigh. The hyperpigmented plaques gradually enlarged over a period of six years, during which the patient was free of rheumatological symptoms but suffered unexplained iron deficiency anemia for which he had oral tonic therapy on a regular basis and received blood transfusion therapy on multiple occasions.

Six years ago, the patient started developing multiple bouts of severe joint pain, swelling, tenderness, warmth, and restriction of mobility, mainly involving the small joints of the hands, wrists, and ankle joints. The arthropathy was bilateral, symmetrical, and had a relapsing-remitting course, worsened by stillness and associated with marked fatigability, unexplained fever, and a worsening flexure deformity of the toes. There was also a progressive increase in the surface areas covered by the hyperpigmented plaques to involve the posterior aspect of the thighs, buttocks, back, and legs. Systemic steroid therapy was introduced but came off short.

Now, the patient presented with a more progressive course with worsening of the indurated lesions, more frequent relapses of arthritis, involving more joints of the body (elbow, neck) with markedly diminished functionality and progressively worsening bone and muscle pain. The patient also reported having multiple nodules scattered over the limbs, persistent bilateral eye pain, and bilateral, symmetrical, ankle swelling.

On examination, the patient had low-for-age stature, with anthropometric measurements of 150 cm height (-2.4 from standard deviation), 96 kg weight, and a BMI of 38 kg/m^2^ (obese -Class II)

**Figure 1:**
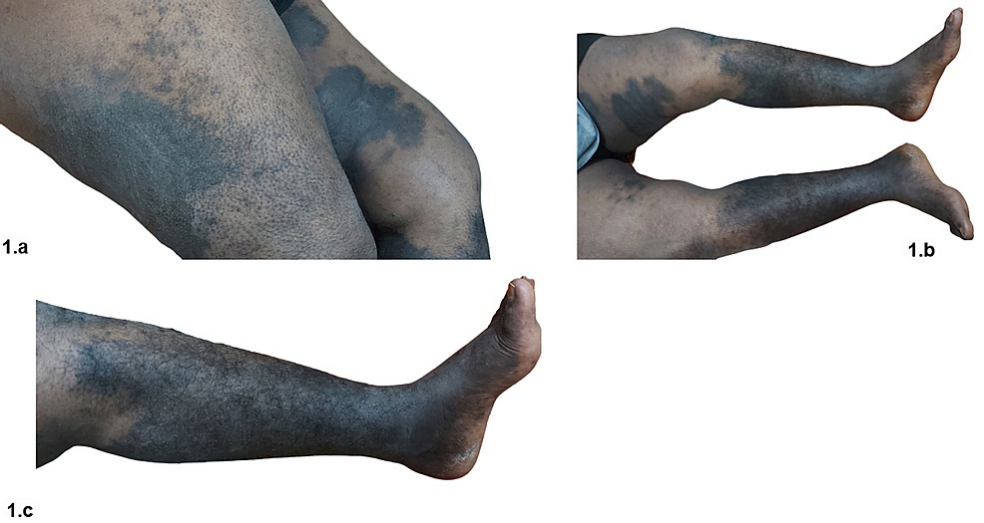
Large, bilateral, irregularly bordered hyperpigmented plaques with hypertrichosis and induration, over the medial and posterior aspect of the thighs (a), anterior, medial, and posterior aspects of the legs (b), with knee and foot sparing (b, c)

A well‑defined, bilateral, hyperpigmented, warm-to-touch, indurated plaques with hypertrichosis present over medial, lateral, and posterior aspects of thighs and legs sparing knees and feet were noticed (Figure 1a, 1b, 1c).

**Figure 2:**
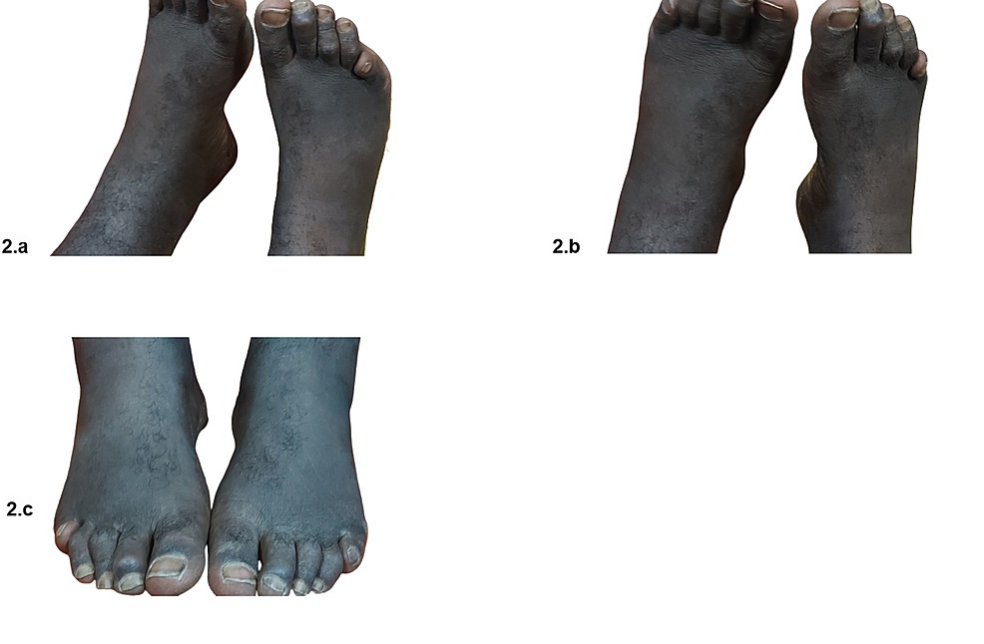
Marked high-arched feet (a, b), fixed flexion deformity of the toes (c), and a mild degree of hallux valgus (c)

The patient also exhibited marked high-arched feet (Figure 2a, 2b), fixed flexure deformity of the toes, and a mild degree of hallux valgus (Figure 2c).

**Figure 3:**
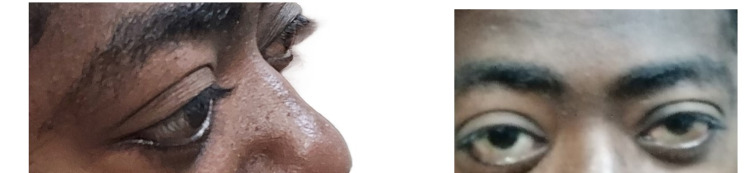
Marked bilateral exophthalmos

Bedside examination also revealed hypogonadism, gynecomastia, multiple rheumatoid nodules over the extensor surfaces of the four limbs, and bilateral, symmetrical, grade 1 pitting lower limb edema. While bilateral exophthalmos was markedly present (Figure 3), fundoscopic examination was normal for both eyes as well as bedside audiometry tests.

Hematological investigations revealed iron deficiency anemia with red blood cell (RBC) microcytosis and hypochromia, 9 g/dL hemoglobin level, 30% hematocrit, and RBC indices of 65 fL mean corpuscular volume, 20 pg mean corpuscular hemoglobin, and 30 g/dL mean corpuscular hemoglobin concentration. Total leukocyte was normal. Iron studies were significantly positive for iron deficiency anemia with serum iron of 14 ug/dL, total iron-binding capacity of 380 ug/dL, transferrin saturation of 3%, and serum ferritin level of 20 ng/mL.

The patients had normal liver, kidney, and thyroid functions in addition to normal blood glucose levels (random/fasting/postprandial). There was also significantly elevated erythrocyte sedimentation rate, C-reactive protein, and anti-nuclear antibody levels with otherwise normal immunological report (Table 1).

**Table 1:** Immunological profile of the patient ANA, anti-nuclear antibody; ANCA, anti-neutrophil cytoplasmic antibody; ESR, erythrocyte sedimentation rate; dRVV, dilute Russell's viper venom.

Test		Result	Comment
ANA		1.6	Positive
ANCA (cytoplasmic)		4.2 U/mL	Negative
ANCA (perinuclear)		0.6 U/mL	Negative
Anti-Smith and RNP		Negative	Negative
Anti-Smith antibody		Negative	Negative
Anti-SSA(Ro) antibody		Negative	Negative
Anti-SSB(LA) antibody		Negative	Negative
Anti-Jo.1 antibody		Negative	Negative
Anti-SCL 70 antibodies		Negative	Negative
Anti-centromere (serum)		Negative	Negative
ADNA (serum)		Negative	Negative
C3 level		148 mg/dL	Normal
C4 level		19 mg/dL	Normal
Anti-cardiolipin IgM		2.0 MPLU/mL	Negative
Anti-cardiolipin IgG		3.1 MPLU/mL	Negative
Beta 2 glycoprotein antibody (IgG)		4.8 U/mL	Negative
Beta 2 glycoprotein antibody (IgM)		3 U/mL	Negative
Lupus anticoagulant	dRVV screen ratio	1.2	Negative
	dRVV normalized ratio	1.03	
	dRVV confirm ratio	1.17	
Coomb’s test (direct)		Negative	Negative
Coomb’s test (indirect)		Negative	Negative
Rheumatoid factor		5 IU/mL	Normal
C-reactive protein		38.4 mg/L	Elevated
ESR	First hour	69 mm	Elevated
	Second hour	100 mm	Elevated

X-rays of wrist, hands, and ankle were normal, the abdominal ultrasound showed splenomegaly, and echocardiography showed mild tricuspid valve regurgitation, right ventricular dilatation, and right ventricular pressure of 56 mmHg.

**Figure 4:**
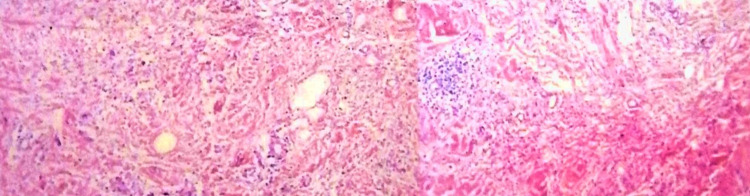
Punch skin biopsies showing dermal fibrosis, diffuse interstitial of lymphocytes, histiocytes, and plasma cells with lymphoid aggregates and proliferated thick-walled blood vessels

Skin biopsy from the indurated hyperpigmented plaques showed thickened collagen bundles with lymphocytic and histiocytic infiltrates in the dermis extending to the subcutaneous tissue with fibrosis with CD68 positivity in dermal perivascular histiocytic infiltrate (Figure 4).

## Discussion

H syndrome is an extremely rare entity that is autosomal-recessively inherited and presents with a variety of multisystemic manifestations and overlapping features with various other conditions. So far, it is estimated that there is a total number of near 100-120 cases worldwide since the first reported in 2008 [1,3,5], with an estimated incidence rate of less than 1 in 1,000,000 [6].

The condition is caused by a mutation in the solute carrier family 29 (nucleoside transporters), member 3 (SLC29A3) gene, which encodes for the hENT3 [4,7]. hENT3 is a nucleoside transporter that functions in transporting nucleosides through a passive sodium-independent process, a crucial step for nucleotide synthesis via salvage pathway [8]. With near 20 mutations found to be affecting SLC29A3 gene, the association of this gene with various clinical conditions has been described recurrently in the previous literature to include genodermatosis, Faisalabad histiocytosis, Rosai-Dorfman disease (RDD), and H syndrome [1,2,7-10]. And though marked interfamilial and intrafamilial clinical variability was noticed on multiple occurrences, reported mutations involving SLC29A3 gene are considered to have no genotype-phenotype correlation [11].

Most of the reported cases of H syndrome were from Asian, Arabian, Persian, Hispanic, and Caucasian ethnicities [5,11-13], And so far, to the best of our knowledge, this is the first case of H syndrome reported in the literature presented in a patient of African ethnicity. And while the patients we are reporting exhibited near (95%) to the common findings of H syndrome described in the literature including hyperpigmentation (68%), hypertrichosis (68%), short stature (49%), hypogonadism (6%), splenomegaly (43%), heart anomalies (34%), hematological abnormalities, arthritis (8%), foot deformity (20%), hallux valgus (20%), flexion contractures of toes (56%), exophthalmos (28%), and gynecomastia [1,11,12], in addition to the characteristic histological finding of H syndrome, genetic diagnosis, which was logistically burdensome to obtain in our low-resource settings, is strongly advised for further confirmation of the diagnosis and for genetic and familial counseling and support.

The differential diagnosis for such concurrent clinical findings is quite a few, and one must rule out common conditions known to be associated with SLC29A3 mutations, with especially RDD and Faisalabad histiocytosis having the most overlapping features with H syndrome [14,15]. Both are to be excluded, by the absence of the characteristic generalized lymphadenopathy in our patient [14,16,17]. In the same way, one could easily exclude POEM syndrome by the absence of polyneuropathy [18], and localized fibrosing disorders (morphea, scleroderma, and regional fibrosis) by the irrelevant immune profile and histological findings of our patients [19].

## Conclusions

H syndrome is a very rare condition with very few reports in the literature, and so far, this is the very first case report of H syndrome encountered in African ethnicity. The phenotypical variation, the vast clinical variability, and the complex overlap of H syndrome with various syndromes and other medical conditions make it one of the very tough cases to pick up during practice. For this, physicians should be very vigilant and sharp-eyed for more accuracy of diagnosis, more effective management, better outcome, and proper counseling and support.
